# Value of Geographical Information Systems in Analyzing Geographic Accessibility to Inform Radiotherapy Planning: A Systematic Review

**DOI:** 10.1200/GO.22.00106

**Published:** 2022-09-19

**Authors:** Varsha Hande, Jessica Chan, Alfredo Polo

**Affiliations:** 1 Applied Radiation Biology and Radiotherapy Section, Division of Human Health, International Atomic Energy Agency, Vienna, Austria; 2 Department of Radiation Oncology, BC Cancer, Vancouver, BC, Canada; 3 Department of Surgery, University of British Columbia, Vancouver, BC, Canada

## Abstract

Vulnerable populations face geographical barriers in accessing radiotherapy (RT) facilities, resulting in heterogeneity of care received and cancer burden faced. We aimed to explore the current use of Geographical Information Systems (GIS) in access to RT and use these findings to create sustainable solutions against barriers for access in low- and middle-income countries.

**MATERIALS AND METHODS:**

A systematic review using the PRISMA search strategy was done for studies using GIS to explore outcomes among patients with cancer. Included studies were reviewed and classified into three umbrella categories of how GIS has been used in studying access to RT.

**RESULTS:**

Forty articles were included in the final review. Thirty-eight articles were set in high-income countries and two in upper-middle–income countries. Included studies were published from 2000 to 2020, and were comprised of patients with all-cancers combined, breast, colon, skin, lung, prostate, ovarian, and rectal carcinoma patients. Studies were categorized under three groups on the basis of how they used GIS in their analyses: to describe geographic access to RT, to associate geographic access to RT with outcomes, and for RT planning. Most studies fell under multiple categories.

**CONCLUSION:**

Although this field is relative nascent, there is a wide array of functions possible through GIS for RT planning, including identifying high-risk populations, improving access in high-need areas, and providing valuable information for future resource allocation. GIS should be incorporated in future studies, especially set in low- and middle-income countries, which evaluate access to RT.

## INTRODUCTION

Health care should be easily available, accessible, acceptable, and of high quality.^1-3^ It should be affordable for all, and thus, equitable.^4^ This is difficult for health care systems in developing countries because of disproportionate distribution of resources, services, patients, and burdens of diseases.^5^

**CONTEXT**

**Key Objective**
To examine published evidence regarding how Geographical Information Systems (GIS) has been used in assessing geographic access to radiotherapy (RT) planning during cancer care across the world.
**Knowledge Generated**
The use of GIS to study access to RT planning was classified into three broad categories: GIS used to describe geographic access to RT, to associate access to RT with health outcomes, and for RT planning.
**Relevance**
GIS can be used to study cancer incidence, risk factors, and uptake or interruption of treatments, and use this information to plan, implement, and monitor targeted health interventions. The technology can compare RT demand and uptake to determine access across regions and income levels. GIS allows the use of prediction models of environmental exposures or changes in resource distribution and their associated outcomes. Through this, GIS reveals previously unidentified factors, trends, and disparities among vulnerable populations and thus, aids in equitable resource redistribution.

Cancer is the second leading cause of mortality^6^ and responsible for one of the largest disease burdens. Adequate management, through targeted screening, early diagnosis, and appropriate treatment, reduces mortality and high health care system costs.^7^ Unfortunately, late-stage presentations are common, especially in resource-strained, low- and middle-income country (LMIC) settings, which confer poor outcomes. This can be due to barriers in accessing health care, difficulties of long treatment schedules, and lack of patient support.^8,9^

Radiotherapy (RT) is an essential component in the cancer control continuum and has a demonstrated population benefit on local control and overall survival.^10,11^ Optimal RT utilization rate (RUR), at any cancer stage, is around 50%.^12^ However, differences between optimal and actual RUR have been described across income settings,^12-18^ leading to detrimental outcomes.

This gap between optimal and actual RUR reflects discrepancies in practice, policies,^19^ and access because of differences in availability across regions, even within one health care system. Actual RUR may be dependent on availability of resources (transport, time, and cost), especially for patients from regional or rural areas, to overcome geographical barriers (long distances, inaccessible roads, and remote areas).^20^ This leads to incomplete treatment,^21^ especially if associated costs^18^ outweigh perceived health benefits.^22^ Thus, geography influences survival^23^ and care^2-6,24^ of patients with cancer.^25^

Geographical Information Systems (GIS) are software packages designed to store, manage, and display spatial information and aid in analysis and interpretation of such data.^23^ In health care, GIS can be used to study basal health indicators and trends among populations and regions, and use this information to plan, implement, and monitor targeted health interventions.^24,26^ In addition to mapping incidence, risk factors, and availability of treatment options, GIS can also run prediction and simulation models on potential environmental exposures, or changes in resource allocation.^26^ GIS reveals trends in access and uptake of interventions, unmasking unforeseen factors affecting vulnerable populations. Consequently, GIS exposes potential health disparities and aids in equitable resource reallocation,^22^ on the basis of need, incidence, and utilization or omission of treatment,^27^ especially useful in LMICs. GIS can also be used to characterize health-related traits, such as economic or social, in specific populations, to build targeted interventions. In high-income countries such as Australia and Canada, GIS is useful to assess how successfully existing RT centers are able to meet community needs, especially among vulnerable sections of society and considering sparsely populated areas and differences in urban/rural balance.

RT services are complex to set up in LMICs as they require large upfront costs for infrastructure and skilled personnel. GIS has a unique potential to aid in RT provision from a geographic perspective and to design optimized networks that improve outcomes through several approaches: supplying RT machines to areas of greater need, reducing distances to RT facilities, implementing national RT infrastructure, etc.

Given its utility in other health care fields,^24,28-32^ we conducted a systematic review analyzing how GIS has been used to examine geographic accessibility to, utilization of, and planning of RT resources.

## MATERIALS AND METHODS

### Search Strategy

A search strategy adhering to the PRISMA statement^33^ was developed with terms relating to GIS, RT, carcinoma, geographic analysis, and spatial analysis (Data Supplement). This search strategy was developed through an iterative process of updating and refining MeSH and keywords on the basis of relevant papers. Electronic searches were conducted on PubMed, EMBASE, Web of Science, and Medline up to 13th September 2021 with no restriction on publication year. Eligible articles already known to reviewers, and studies found in the reference sections of papers were also considered.

### Inclusion Criteria

This review included all studies that explicitly stated the use of GIS in their methods section to explore availability of RT services among patients with cancer (Table 1). All cancer types and geographic factors, such as distance, variation, time, practices, utilization, distribution, access, density, and deprivation, were considered. There was no restriction regarding country, sample size, age, sex, or demographics. Ecological, cohort, and case-control studies were included.

**TABLE 1:**
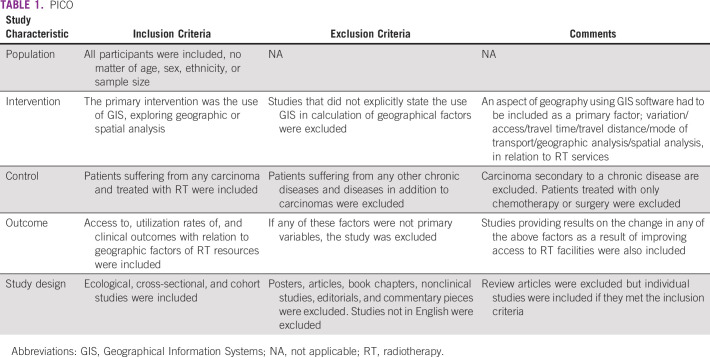
PICO

### Exclusion Criteria

Studies not using GIS, exploring treatment modalities other than RT, or not analyzing cancer patient samples were excluded. Patients with carcinoma because of, or in addition to, concomitant diseases were excluded. Posters, articles, book chapters, nonclinical studies, editorials, and commentary pieces were excluded. For reviews, individual studies were obtained, and reviews were excluded. Full-length articles not in English were also excluded.

### Study Identification and Data Collection

Two reviewers (V.H. and J.C.) independently evaluated studies from titles and abstracts. Eligible studies were then evaluated by both authors independently through full text review. The following data from chosen studies were extracted: reference, study year, design, country, sample size, population source, cancer type, and main results. Because of the heterogeneity of the relevant studies, a narrative synthesis was done. Individual study quality appraisal was conducted against a predetermined selection criterion. Studies were then classified into three themes: (1) GIS used to describe geographic accessibility to RT; (2) GIS used to associate geographic accessibility to RT with outcomes; and (3) GIS used for RT planning. Studies could be classified into more than one theme where appropriate.

## RESULTS

**FIG 1:**
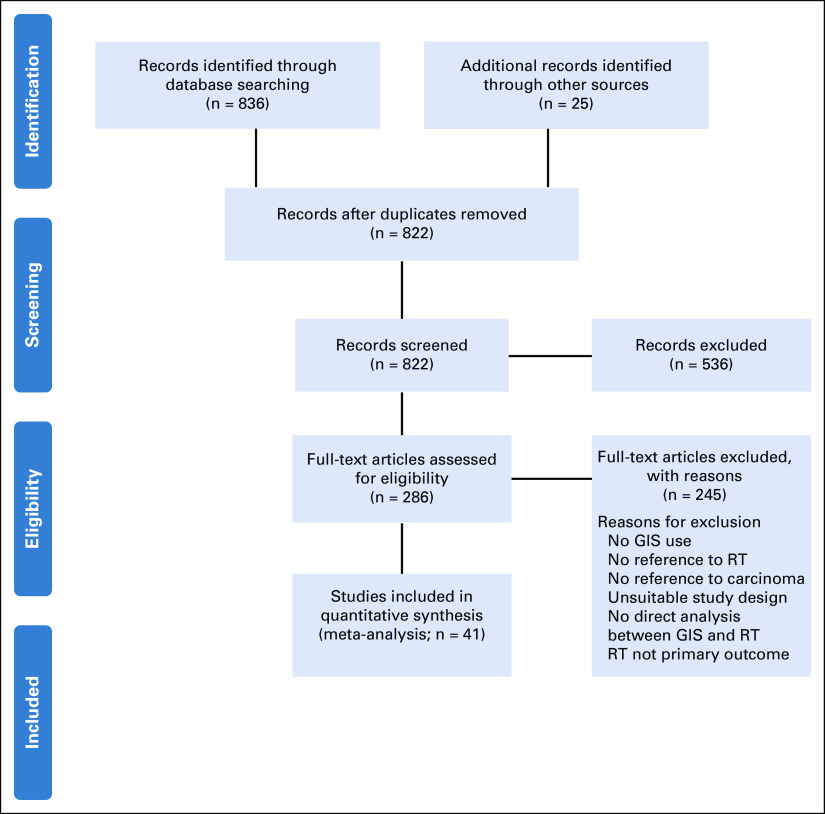
PRISMA flow diagram for systematic reviews. GIS, Geographical Information Systems; RT, radiotherapy.

As shown in Figure 1, 817 studies were screened, excluding 39 duplicates. Of these, 536 were excluded for the following: unavailability of English version, editorials/commentaries, full text irretrievable, and no geographical aspect studied (Data Supplement). Full reviews of the remaining 281 studies were done, and 41 studies were included in the review.

Thirty-eight of the 41 included studies were from high-income countries, and two from upper-middle–income countries. Six studies were from Australia, two from Brazil, nine from Canada, one from Italy, one from Norway, one from the Pacific Island Countries and Territories, seven from the United Kingdom, and 14 from the United States. All studies were published from the year 2000 and onward. Thirty-four studies were cross-sectional, three were retrospective cohorts, one was a historical prospective cohort, and three were ecological studies. Sample sizes ranged from 112 to 353,989. Sixteen studies examined multiple cancer types, 14 examined breast cancer, three studied prostate cancer, two studied patients with lung cancer, and several analyzed a singular tumor.

The 41 studies were classified into three broad themes: GIS used to describe geographic accessibility to RT, GIS used to analyze health systems or patient outcomes, and GIS used for RT planning, summarized in Table 2.

**TABLE 2:**
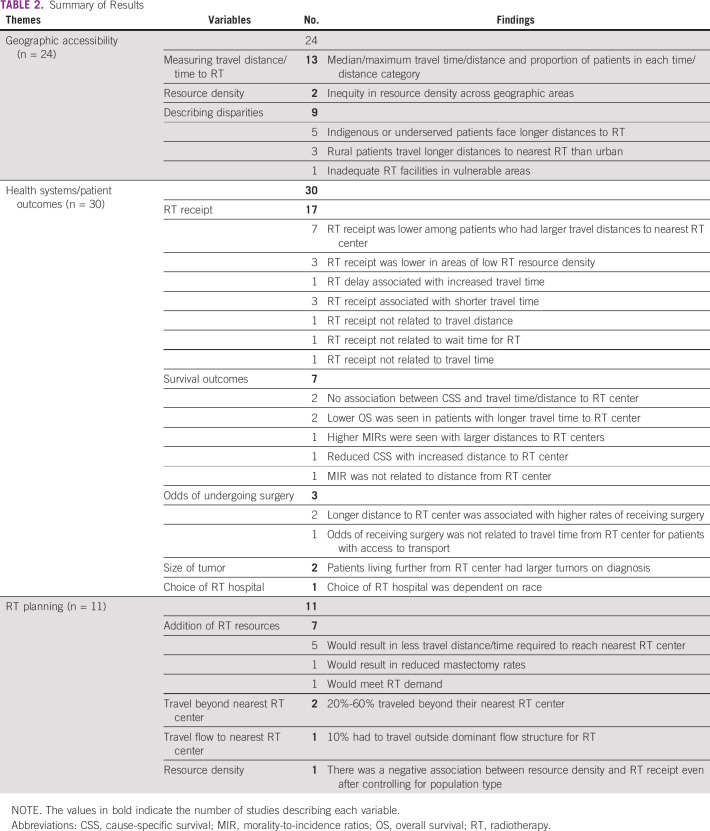
Summary of Results

### GIS Used to Describe Geographic Accessibility to RT

Twenty-four studies used GIS to describe geographic accessibility to RT (Table 3), measured via travel distance^5,34-49^ and travel time^3,4,20,22,35,36,45,48,50-55^ to nearest RT centers. These were calculated on the basis of travel by road,^3,4,20,22,34-36,38,40,42-57^ air,^36,58^ or by Euclidean distance.^5,37,39,41^ Several studies calculated geographic catchment areas and defined vulnerable population groups to describe access.^22,35,41,43,46,51^ Various studies used a cutoff radius of 50 km for catchment areas per RT center, but there was no consensus regarding optimal values. Yao et al^56^ used location of county at diagnosis to define vulnerable populations. Aneja et al^57^ and Lin et al^47^ both calculated density of RT resources in various hospital service areas.

**TABLE 3:**
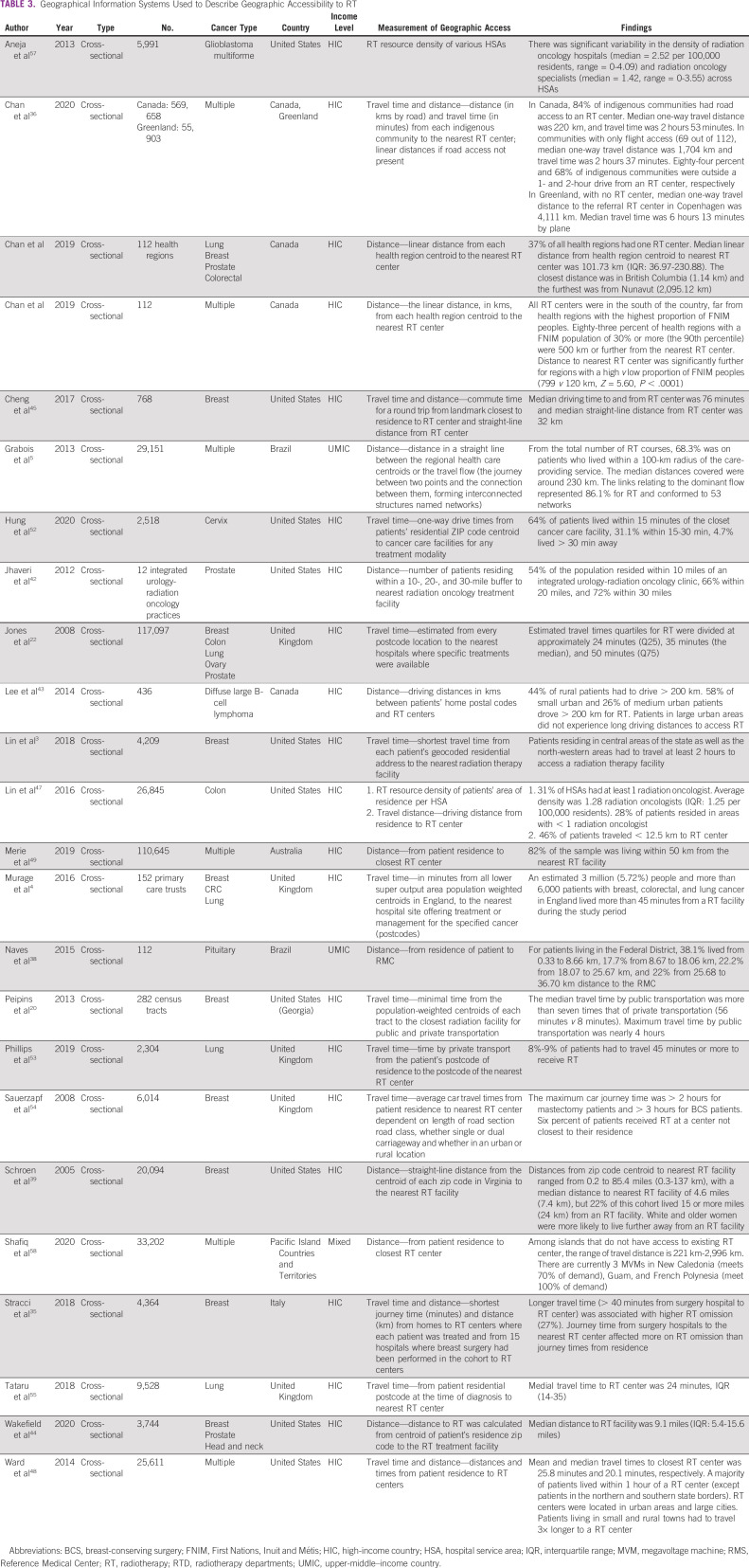
Geographical Information Systems Used to Describe Geographic Accessibility to RT

Notably, 18 studies used GIS to describe disparities in geographic accessibility to RT.^3,5,20,34,36,37,39-44,48,56,58^ Four studies^3,38,43,48^ found that rural-residing patients had to cover larger road distances to the nearest RT center, compared with urban-residing patients, and five studies reported that Indigenous or underserved populations faced inequitable RT access.^36,37,41,56,58^ Yao et al^56^ and Shafiq et al^58^ found that, relative to surrounding areas, RT facilities in areas with vulnerable were inadequate.

### GIS Used to Analyze Health Systems or Patient Outcomes

Thirty studies used GIS to associate geographic measures to patient and/or health system outcomes (Table 4), such as incidence, latency for diagnosis, tumor size at diagnosis, use of primary surgery, RT utilization/interruption, delay in receiving RT, choice of hospital to receive RT, density of RT resources, cause-specific survival, disease-free survival, overall survival, and mortality to incidence ratios. Most studies used multiple outcomes. Common covariates analyzed were age, sex, tumor site/stage/sequence, race/ethnicity, and socioeconomic status.

**TABLE 4:**
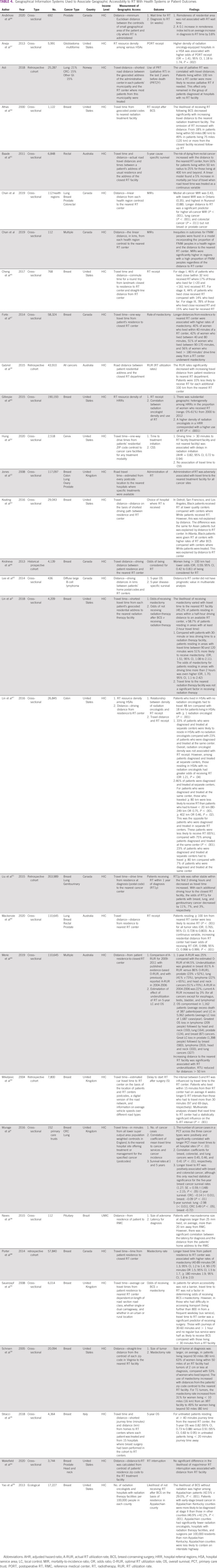
Geographical Information Systems Used to Associate Geographic Accessibility to RT With Health Systems or Patient Outcomes

RURs decreased with increasing distances to nearest RT centers. This was demonstrated for palliative RT,^40,51^ adjuvant RT for breast cancer,^3,4,34,35,39,45,46,49-51,59,60^ rectal cancer,^4,22,40,49,60,61^ lung cancer,^4,22,34,40,49,51,60^ colon cancer,^47,49^ and prostate cancer.^22,49,60^ Regional differences were noted in Norway, Australia, the United Kingdom, and the United States.^3,34,40,50,56,62,63^ Other covariates such as sex,^22,34,38,40,47,49,51,61^ age,^3,22,34,35,38-40,44,46,47,49-51,54,57,60,61^ household income,^40,43,44,47,57^ deprivation level,^3,4,35,49-52,54,60,64^ and tumor type^3,22,35,40,44,49-51,61^ also influenced RT receipt. Higher density of RT-equipped hospitals^57^ and radiation oncologists^47,65^ was associated with higher odds of RT receipt in the United States,^57,65^ but one study^47^ reported that this only held true for patients diagnosed and treated at the same RT center.

Omission or delay in RT administration was studied using GIS. Multiple studies examined factors leading to postoperative RT delay for breast cancer.^3,44,49,50,54,56,59^ Longer distance to nearest RT centers was an independent predictor for delay RT initiation in cervical^52^ and prostate^66^ cancers, and for postoperative RT in breast cancer.^56^ Type of surgery received by patients with breast cancer was also geographically influenced; studies showed a correlation between longer distances or travel time to nearest RT facilities and higher mastectomy rates.^3,39,67,68^ However, a study in England^54^ specified that longer travel time was only predictive of mastectomy among populations facing difficult access to community transport services. For both outcomes, covariates such as age, comorbidities, income, and tumor size were influential. Tumor size at diagnosis was not associated with geography,^38,39^ but more so with sex and age. Conversely, a study^44^ exploring breast, prostate, and head and neck cancers found that distance to RT facility was not associated with RT interruption.

Importantly, GIS was also used to link geography with treatment results through direct patient survival data^4,35,43,49,52,61^ or mortality-to-incidence ratios (MIR).^37,41^ By cancer type, the authors found a relationship between travel time and survival for lung^4,41^ and colorectal^4,41,61^ cancers, but not for breast cancer.^4,35,41^ Disparately, distance to RT was predictive for higher MIR in prostate cancer^41^ or for survival in lymphoma.^43^ Using aggregated data, Chan et al^37,41^ found that increasing distance to nearest RT centers was independently associated with poorer all-cancer MIRs.

### GIS Used for RT Planning

Eleven studies analyzed the effects of addition or relocation of a RT center or supporting facilities in areas of need on RT utilization (Table 5). Chan et al^36^ explored travel saved over 10 years by addition of new RT centers among Indigenous populations in Arctic areas of Canada and Greenland. In scarcely populated areas such as Australia, adding one RT center significantly reduced travel distance and time^69^ and increased the RUR by 14% but only for patients who are living within the closest distance category to an RT center.^70^ The effect was similar in Canada.^51^ An additional RT center in rural Virginia reduced mastectomy rates for breast cancer, and RURs reached levels similar rates to surrounding urban population.^39^ In Italy, adding RT centers significantly reduced the proportion of patients with breast cancer who omit postoperative RT.^35^ Shafiq et al^58^ showed that establishing 26 new megavoltage machines in areas of highest need in Pacific Island Countries and Territories on the basis of 2040 modeled cancer incidence met 90% of the RT demands, compared with 14% with current resources. Aggarwal et al^71^ found that 21% of patients with prostate cancer traveled beyond their nearest RT center for treatment in the United Kingdom. Twenty-one percent of the sample preferred to bypass their nearest RT center to one further away for treatment, but this was inversely associated with increasing travel time. Similarly, Ward et al^48^ reported that 60% of urban-residing patients in the United States bypassed their nearest RT center and chose to travel an additional average 27.9 minutes, but rural-residing patients did not have similar access to multiple RT centers.

**TABLE 5:**
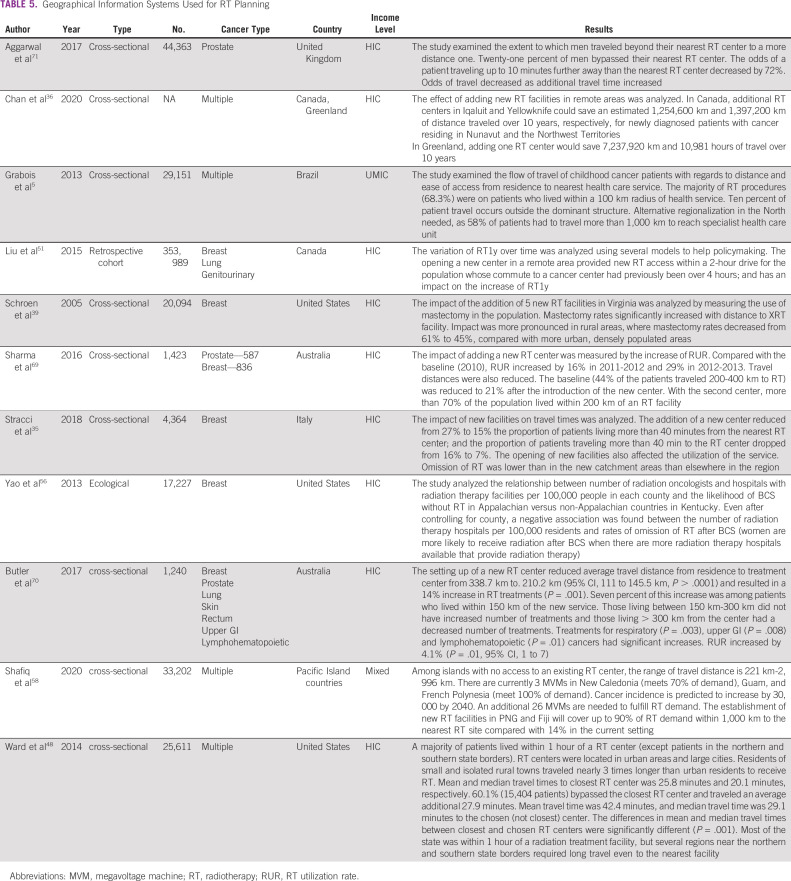
Geographical Information Systems Used for RT Planning

## DISCUSSION

RT access depends on multiple factors, including geography, and has an impact on RUR and cancer outcomes. Thus, there is a need to trace patterns of use and reallocate resources by identifying low RUR populations and regions. Our systematic review demonstrated that GIS is a powerful tool that has only recently been applied to the field of RT to describe geographic accessibility, associate accessibility with cancer outcomes, and assist in RT planning.

When describing geographic accessibility to RT, geospatial techniques are helpful in not only quantifying access, but also in highlighting disparities that were otherwise hidden. It also provided a methodology to analyze and understand patterns of RT access, not only by geography, but also by population group within a region, such as Greenland,^36^ France,^72^ Ecuador,^73^ and the United States.^74,75^ These techniques are relevant in health policy to advocate for improved access among underserved regions. These techniques can be applied in LMICs, where infrastructure for data capture and assimilation is underdeveloped.

In quantifying geographic accessibility and linking this with other health care data sets, GIS allows an unprecedented ability to model its impact, among other covariates, on cancer outcomes. Main outcomes examined in our review were incidence,^4^ latency for diagnosis,^38^ tumor size at diagnosis,^38,39^ use of primary surgery,^3,39,54,67,68^ RT utilization,^22,34,40,45-47,49,51,57,59,60,65^ delay in RT,^3,44,50,52^ cause-specific survival,^52,61^ disease-specific survival,^43^ overall survival,^4,35,43,49,61^ and MIRs.^37,41^ Most studies in our review reported poorer outcomes with increased distance to RT centers. However, many other covariates also played an important role, highlighting the importance for future studies to incorporate geography within multivariate analyses to uncover its unique influence.

GIS was also used for RT planning. Several studies used GIS to calculate changes in travel times and distances with the addition of RT centers in areas of need. Mean travel times and distances reduced,^36,70,71^ leading to improved outcomes, including increased RUR,^51,58,69^ reduced RT omission,^5,35,56^ and reduced mastectomy rates.^39^ Many standalone components, such as single machine units, were found to have similar standards of care as larger or central departments.^76^

Although the existing evidence on GIS and RT is promising, GIS remains underused, particularly as a tool to model strategies to improve RT utilization. Further studies are required to compare the cost-effectiveness of placing RT units in regions with poorer geographic accessibility with alternative models of health service delivery, such as visiting specialists or teleoncology via a hub-and-spoke model.^35,36,77-80^ GIS could be used as a surveillance mechanism to identify prevalence rates and match them to treatment omission trends, enabling strategic placement of required hub-and-spoke centers for optimal service delivery. Telemedicine models mitigate geographic barriers among vulnerable populations and allow overburdened health care sectors to function. However, this applies more to screening and diagnosis, rather than treatment or palliative support. Thus, robust RT treatment infrastructure to assist patient transportation and accommodation is crucial.^3,5,20,39,44,61^

There is a dearth of evidence regarding GIS use in LMICs, and for vulnerable populations in high-income countries. Indeed, all included studies in our review were from high-income or upper-middle–income settings. Since 70% of all cancer deaths occur in LMICs,^81^ utilization of GIS to trace risk factors, incidence, utilization, and thus, reallocation or remodeling of treatment resources in these settings is required. However, because of limited resources, cancer registration data from LMICs is incomplete or low quality. A study demonstrated that, despite poor data availability, GIS helped trace childhood cancer trends in the West Bank. GIS was successfully used to match demand to health care resources in the context of HIV across Africa,^23^ infant mortality in Ghana,^82^ cervical cancer screening services in Nigeria, and cardiac diagnostics in Thailand.^32^ However, there is an important need for more data and training in spatial thinking, particularly in LMICs.^30^ Apart from a lack of data, the leading challenge in LMICs is the need to modify spatial access models to match local settings. Advantageously, GIS can operate remotely. There is immense potential in using GIS to implement feasible solutions (such as strategic placement of telemedicine clinics or targeted improvement of transportation services), to reduce LMIC morbidity and mortality.

Although this review focused on the application of GIS in RT, this is only one component of comprehensive cancer management. Geographic accessibility to other treatment modalities and services are equally important. GIS has been used to study geographic accessibility to mammography,^83-87^ chemotherapy,^72^ surgical care,^88^ and palliative care.^89^ However, to adequately address disparities, future studies should describe trends of all components, from screening through to treatment and survivorship.

There were several limitations to our review. As the use of GIS in public health, and specifically, in RT planning resources is an elementary field, research is limited, and the evidence is evolving. We chose studies that explicitly stated the use of GIS to calculate geographical measures, most commonly distances and travel times, in their methods section. Studies describing similar results but not mentioning GIS had to be omitted.^90-93^ The search terms might not have been all-encompassing, as terminology in this sphere is evolving. To follow the methods of a systematic review, we defined the aspect of GIS through explicit search terms. As there is not yet a standardized format to report GIS use, studies reported this in heterogeneous ways. Accordingly, these studies had to excluded, and we recognize this as a selection bias. We hope that this review acts as a first step in the standardization process of the reporting the use of GIS in health research.

Since the methods of analysis of included studies were heterogeneous, a narrative synthesis was performed, and a risk-of-bias appraisal could not be performed. Individual study quality was assessed against the selection criteria to overcome this. This reinforces the need for a standardized quality appraisal method for such studies that are heterogeneous in their materials and methods. Risk of bias was not assessed owing to heterogeneity between studies. Studies were mostly from high- and middle-incoming settings, so extrapolations must be made with care to LMIC settings. By excluding studies in languages other than English, some relevant studies, especially from LMICs, might have been excluded from our analysis. Most studies in this review were cross-sectional, so causality could not be determined. One of the studies was ecological, so findings cannot be ascribed to the individual. The study samples varied across time periods, ages, socioeconomic statuses, cancer type, and health care need. Homogeneous conclusions drawn from these analyses, therefore, need to be carefully considered according to study specifics.

In conclusion, although GIS has only recently been applied to research RT, we demonstrated that it has significant potential to perform a wide array of functions and ultimately inform policymaking. The ability to quantify geographic accessibility empowers the linkage with other existing health data sets, allowing visualization and understanding of geographical influences on cancer care to inform RT resource allocation. It is widely applicable and adaptable to any setting or population, and easily scalable to regional, national, or international levels. Future studies should model alternative health service delivery to improve geographic accessibility to RT, build evidence banks for LMIC populations, and explore the use of GIS across the entire process of cancer care.

## Data Availability

Data sharing is not applicable to this article as no new data were created or analyzed in this study.
